# Antimicrobial Drug Resistance in Poultry Production: Current Status and Innovative Strategies for Bacterial Control

**DOI:** 10.3390/microorganisms11040953

**Published:** 2023-04-06

**Authors:** Raquel Abreu, Teresa Semedo-Lemsaddek, Eva Cunha, Luís Tavares, Manuela Oliveira

**Affiliations:** 1 CIISA—Centro de Investigação Interdisciplinar em Sanidade Animal, Faculdade de Medicina Veterinária, Universidade de Lisboa, Avenida da Universidade Técnica, 1300-477 Lisboa, Portugal; 2 Laboratório Associado para Ciência Animal e Veterinária (AL4AnimalS), 1300-477 Lisboa, Portugal

**Keywords:** poultry production, microbiota, antibiotics, antibiotic alternatives, antimicrobial resistance, food safety

## Abstract

The world population’s significant increase has promoted a higher consumption of poultry products, which must meet the specified demand while maintaining their quality and safety. It is well known that conventional antimicrobials (antibiotics) have been used in livestock production, including poultry, as a preventive measure against or for the treatment of infectious bacterial diseases. Unfortunately, the use and misuse of these compounds has led to the development and dissemination of antimicrobial drug resistance, which is currently a serious public health concern. Multidrug-resistant bacteria are on the rise, being responsible for serious infections in humans and animals; hence, the goal of this review is to discuss the consequences of antimicrobial drug resistance in poultry production, focusing on the current status of this agroeconomic sector. Novel bacterial control strategies under investigation for application in this industry are also described. These innovative approaches include antimicrobial peptides, bacteriophages, probiotics and nanoparticles. Challenges related to the application of these methods are also discussed.

## 1. Introduction

The human population is continually increasing, rendering food security a major concern; thus, it is necessary to ensure that food production systems can support this population increase [1]. Animal food products, including meat, play an important role in the human diet. The demand for this foodstuff is on the rise, and meat consumption has increased more than 4-fold in the last 50 years [2].

Nowadays, poultry is one of the most consumed meats worldwide, being the second most produced and consumed meat in the European Union (EU) after pork [3]. In addition, global meat production has increased over the years [2]. From a global perspective, and according to the FAO, in 2020, the production of poultry meat represented almost 40% of global meat production [4]. Consequently, there has been a global shift towards intensive farming systems in which infections, including zoonosis, are transmitted more easily, affecting animal health and productivity [2,5].

Along with the apprehension related to food safety, this increase leads to concerns regarding production sustainability and safety. The production of animal-derived products have inherent impacts to One Health, such as an increase in greenhouse gases, the contamination of drinking water, environmental contamination, the dissemination of antimicrobial drug resistance, and the emergence and re-emergence of zoonotic diseases [6,7]. The production of sufficient amounts of food for the global population is one of the major current challenges [7].

Due to the increasing concentration of animals in intensive farms and the use of conventional antibiotics to safeguard the health of animals and animal products, antimicrobial resistance has developed and spread, which has led to a global public health concern. This review aims to focus on the role of poultry production in the development of AMR and the main bacterial pathogens that affect poultry, and to discuss the potential role of innovative antimicrobial compounds as an alternative or complementary strategy to the use of conventional antibiotics and, consequently, for the reduction and dissemination of AMR between animals, humans and the environment in a One Health Approach.

## 2. Antimicrobial Drug Resistance

### 2.1. Global Scenario

Antibiotics are natural, semisynthetic or synthetic substances, which interfere with the growth or survival of bacterial microorganisms, and are used to prevent or treat the associated infections [8,9]. Although traditional antimicrobial compounds have been recognized for thousands of years since their discovery by ancient civilizations, it was only in 1928 that the first antibiotic, penicillin, was developed by Alexander Flemming [8].

**Figure 1:**
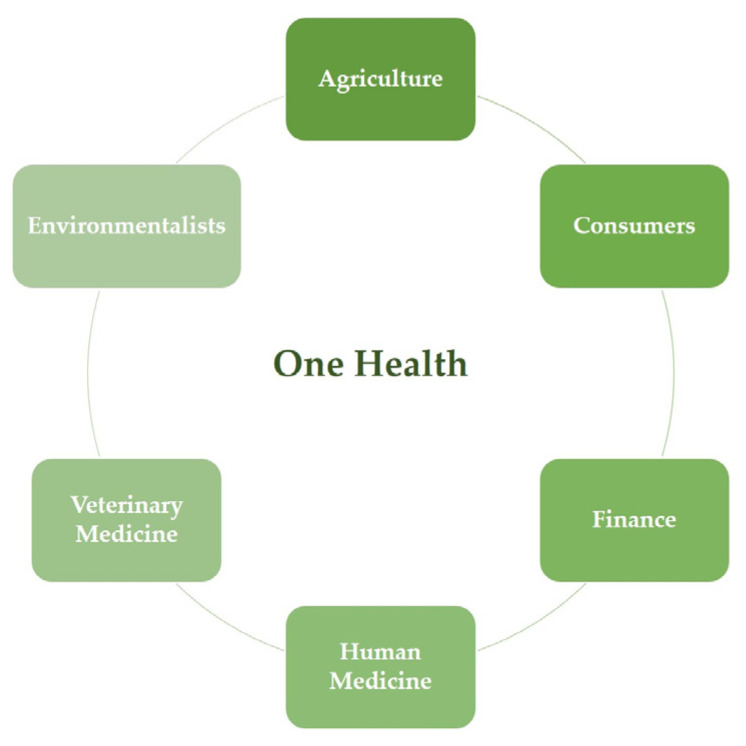
Schematic representation of the coordination between different groups required for a One Health approach.

The advent of antibiotics revolutionized medicine due to their ability to combat bacterial infections, allowing an increase in the average life expectancy of humans and animals, the control of infectious diseases and the reduction in morbidity and mortality, while also contributing to food safety [8,9]. Unfortunately, due to the extensive use of these compounds, multidrug resistant (MDR) microorganisms have emerged and disseminated, which is currently a global concern [10]. If the rate of development of MDR bacteria continues to increase, it is estimated that in 2050 the mortality rate caused by resistant bacterial infections will exceed the mortality rate caused by cancer [11]. In 2000, the World Health Organization (WHO) classified antimicrobial drug resistance (AMR) as a global public health concern. As such, it is urgent to find strategies for the control and mitigation of these strains [11,12]. In 2015, the World Health Assembly (WHA), which is the decision-making body of the WHO, adopted a global action plan focused on AMR based on five objectives: improve awareness of antimicrobial drug resistance; strengthen knowledge about it through surveillance and research; reduce the incidence of infection by effective sanitation, hygiene and infection prevention measures; optimize the use of antimicrobials in human and veterinary medicine; and increase investment in the development of new medicines, diagnostic tools and vaccines, taking into consideration the necessities of all countries. This action plan highlights the need for an effective One Health approach to tackle this issue and requires coordination among several sectors and groups, including human and veterinary doctors, farmers, economists, environmentalists and informed consumers [13] (Figure 1).

To help control AMR dissemination, the European Medicine Agency (EMA) developed a categorization of the conventional antibiotics used in veterinary medicine in order to promote their responsible use, focusing on the protection of public and animal health. As such, antibiotics were classified as category A (“Avoid”), which includes antibiotics that are not authorized in veterinary medicine; category B (“Restrict”), which includes critically important compounds for human medicine for which use in animals should be restricted; category C (“Caution”), which includes antibiotics for which alternatives in human medicine generally exist and can be applied in the veterinary settings in the absence of alternatives belonging to category D; and category D (“Prudence”), which includes the antibiotics that should be used for first-line treatments in animals [14].

### 2.2. Antibiotics in Poultry Production

Antibiotics have been used in animal production for over fifty years as therapeutic and metaphylactic/prophylactic agents or as growth promoters [15]. The efficacy and cost-effectiveness of the majority of these compounds led to their indiscriminate usage [16]. Consequently, the misuse and overuse of these antimicrobials promoted the establishment of microbial reservoirs carrying AMR determinants in livestock, including poultry. As some of the antimicrobials applied to animals are the same as those administrated to humans, AMR dissemination poses a serious threat to the effective treatment of serious bacterial infections in humans, leading to higher medical costs, prolonged hospital stays and increased mortality [17,18,19].

Antimicrobial growth promoters (AGPs) started being applied in 1951, when the United States (US) Food and Drug Administration (FDA) approved the use of antibiotics as animal additives without prescription, followed by European Union (EU) countries, which approved their own regulations on the use of those substances in animal production [18]. AGPs are antibiotics administrated at subtherapeutic doses, aiming to modify the animal’s intestinal microbiota to attain a better performance. AGP dissemination contributes to selecting intestinal bacteria, reducing competition for nutrients and improving animal growth rates. Some authors defend these benefits, arguing that they are important in the early stages of production or that they are useful in the presence of sub-optimal hygiene conditions [20], while others report that they increase productivity, highlighting the importance of good husbandry in animal production [19].

AGP use has contributed to the evolution and spread of AMR in intestinal microbiota [5,21], prompting some countries to ban their application in animal production. Sweden was the first country to prohibit the inclusion of AGPs in animal feed in 1986. In 2006, the EU banned the use of 25 AGPs from animal production. Moreover, EU’s decision to ban AGPs has been adopted by several other countries, such as Mexico, New Zealand and South Korea. On the other hand, the USA, Australia, Japan and Canada implemented laws to partially ban or exclude some antibiotic-derived additives [22]. In fact, some important human medicine antimicrobials have been prevented from being used as AGPs in the US since 2016 [19]. Despite these actions, antibiotics are still relevant for the prevention and treatment of bacterial infections, contributing to animal welfare and to the reduction in zoonotic diseases [5,15,19].

### 2.3. Development of Antimicrobial Drug Resistance

Antimicrobial drug resistance relates to the capacity of a microorganism to survive the inhibitory or killing activity of an antimicrobial compound [10]. This phenomenon has been reported since the discovery of antibiotics [12]. When an antibiotic is administrated, susceptible bacteria are eliminated, favoring the selection of resistant strains. These strains become the predominant bacterial population, allowing the transmission of genetic resistance determinants to clonal descendants, to other isolates of the same species, or even to members of other bacterial species. This phenomenon occurs either in commensal or pathogenic bacteria from humans, animals and the environment [9].

There are two main pathways associated with the evolution and development of antimicrobial drug resistance. The first is related to resistance mediated by pre-existing phenotypes in natural bacterial populations. During the evolutionary process, bacteria accumulate genetic errors in existing genes (present in the bacterial chromosome or in plasmids) and transfer those genetic determinants responsible for innate/natural or intrinsic resistance to progeny cells via vertical gene transfer (VGT) [23]. The second scenario refers to acquired resistance, which may develop via a direct pathway, which involves gene mutations, or an indirect pathway, by the acquisition of DNA fragments coding for resistance (namely, transposons, integrons, phages, plasmids or insertion sequences) by horizontal gene transfer (HGT) mechanisms that may occur between the same or different bacterial species. HGT takes place via either conjugation, transformation or transduction [19,23,24]. VGT and HGT can occur in a variety of settings [19]. As such, farms in which animals and vegetables are produced can act as reservoirs of antibiotic resistant bacteria as the food chain comprises distinct ecological niches, including those in which antibiotics are used and bacteria coexist [25].

### 2.4. Transmission of Antimicrobial Drug Resistance

Drug resistance can disseminate along the food chain through direct or indirect contact between the different actors and settings, both of which are also considered routes of transmission for zoonotic diseases. Direct contact occurs when humans come into contact with resistant bacteria present in animals or in their biological products such as urine, feces, blood, saliva and semen. Occupational workers, such as veterinarians, farmers, abattoir workers and food handlers, and others who have contact with them, have a higher risk of being colonized or infected with resistant strains. At present, it is well established that occupational workers and their families are an entryway for resistant bacteria into the community [9,26]. Alternatively, indirect contact can also lead to infection, and includes the handling and consumption of contaminated food products, such as meat and eggs, in the case of the poultry industry [17,19].

Additionally, a large proportion of antibiotics are not totally degraded, nor are transformed into inactive compounds by animals and humans, and retain their activity after being excreted in urine and feces. The active antibiotic, related metabolites or degradation products, named antibiotic residues, can accumulate in soils, wastewater and manure, causing profound impacts [9,17]. Hence, the dissemination of antibiotic-resistant bacteria and antibiotic residues via food and animal waste turn the environment into an important reservoir of antimicrobial drug resistance [9,27]. In fact, it is known that the disposal of manure from animal pens has a significant role in the promotion of HGT of resistance genes among soil bacteria. This way, natural soil can also play a role as a reservoir of resistance determinants [24]. In addition to commensal and environmental bacteria, foodborne pathogens also carry AMR genes [28].

## 3. Important Pathogens in Poultry Production

The presence of a wide variety of microorganisms is surveilled in several food producing animals due to their importance to public health, namely in broilers and laying hens. The pathogens most relevant in this industry, and associated with antimicrobial drug resistance, include *Salmonella enterica*, *Campylobacter* spp. (specially *C. jejuni*), *Escherichia coli*, *Enterococcus* spp. and methicillin-resistant *Staphylococcus aureus* (MRSA) [29,30].

### 3.1. Salmonella Enterica

*Salmonella enterica* causes foodborne enteric disease worldwide, representing the second most commonly reported zoonotic pathogen in the EU [31,32]. It is responsible for disease outbreaks associated with significant morbidity and mortality [33], and up to 25% of human *Salmonella* outbreaks, illnesses and hospitalizations are related to poultry sources [32,34].

**Figure 2:**
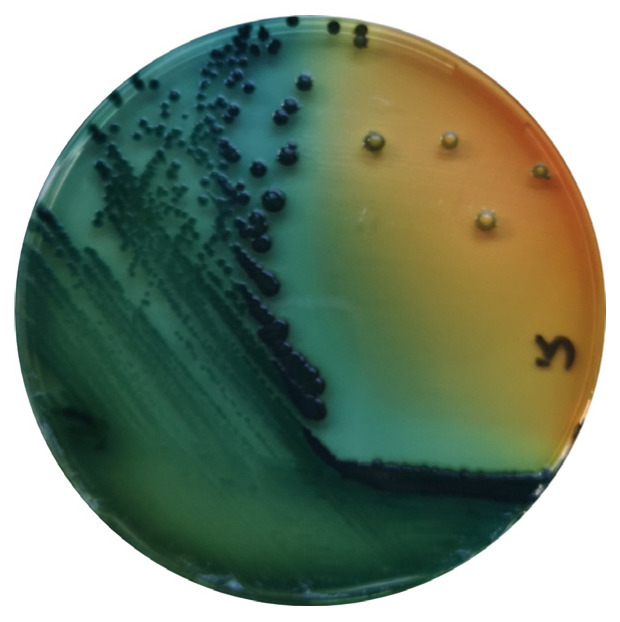
*S. enterica* in Hektoen agar (Oxoid, Hampshire, UK).

*Salmonella* are gram-negative, facultative anaerobic bacteria belonging to the Enterobacteriaceae family, and are considered commensals of the gut microbiota of mammals, birds, reptiles, amphibians, fish and shellfish [35] (Figure 2).

*S. enterica* includes more than 2650 serovars [36], in which several have been previously described as contaminants of poultry meat and eggs, representing a serious concern for public health [37].

In poultry, diseases promoted by *S. enterica* are divided into three conditions: fowl typhoid (promoted by *S. enterica* subsp. *enterica* serovar Gallinarum biovar Gallinarum), pullorum disease (by *S. enterica* subsp. *enterica* serovar Gallinarum biovar Pullorum) and avian paratyphoid, which results from infection caused by other *S. enterica* serovars, including *S.* Enteriditis, *S.* Typhimurium and *S.* Infantis [30]. These bacteria can be transmitted via both vertical (from infected breeders) and horizontal (from other birds in a flock or from the environment) routes [33]. Young poultry are particularly susceptible to gastrointestinal tract (GIT) colonization by *S. enterica*. Its excretion in feces may result in the contamination of the environment and the infection of nearby birds [32,35]. Moreover, poultry meat contaminated with digesta during slaughter is a major risk to public health [32,38].

*S. enterica* can be transmitted from animals to humans through the consumption of contaminated animal derived products, such as meat, and of other foodstuffs contaminated with fecal matter, or through direct or indirect contact with colonized animals or contaminated water. This agent is considered moderately resistant to certain environmental conditions, such as freezing, acidic pH and dehydration, which contribute to its high transmissibility. When infecting humans, *Salmonella* attaches and colonizes the intestinal columnar epithelial cells, resulting in fever, nausea, diarrhea, vomiting and abdominal pain. The disease is usually self-limiting in healthy adults, but it can lead to septicemia and death in severe cases, especially in children, the elderly and immunocompromised patients [33,35]. In these cases, antimicrobial treatment is advised, and fluoroquinolones, macrolides and third-generation cephalosporins can be used to treat *S. enterica* infection [35].

Several studies comprising the evaluation of the antimicrobial drug resistance profiles of more than 4000 isolates of *Salmonella* spp. were revised by Saraiva et al., 2022 [30]. Higher frequencies of resistance were observed towards nalidixic acid, amoxicillin, ampicillin, erythromycin, penicillin G, sulfamethoxazole and tetracycline, while higher susceptibilities were associated with the aminoglycosides spectinomycin and gentamicin.

In the case of paratyphoid *Salmonella*, multidrug resistance is a concern because it can lead to treatment failure. The most common resistance patterns associated with this pathogen include important therapeutic antimicrobial classes used in human medicine, such as penicillins, tetracyclines, cephalosporins and fluoroquinolones, and this association represents a public health concern [30].

### 3.2. Campylobacter *spp.*

**Figure 3:**
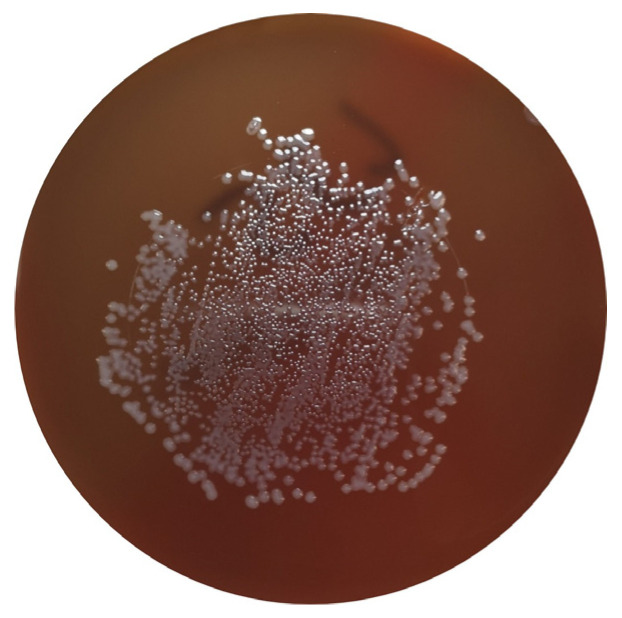
*C. jejuni* in Columbia Agar + 5% sheep blood (bioMérieux, Marcy-l’Etoile, France).

*Campylobacter* spp. are ubiquitous bacteria that can be found in various environments, including soil and water, and as commensals of the GIT of poultry. Despite this, they can cause disease in animals and humans and constitute an important cause of foodborne diseases worldwide [39,40,41]. This bacterial genus can be responsible for acute bacterial diarrhea, which is mainly caused by *C. jejuni* and *C. coli*. Although other sources can be responsible for human infection, poultry products are considered the predominant source of human campylobacteriosis [33] (Figure 3).

*Campylobacter* spp. can be introduced in the production farms by wild animals, pests or humans. When infecting poultry, it colonizes the animal’s intestine, invades the intestinal epithelium and multiplies rapidly in the intestinal mucus, avoiding clearance and persisting in the animal’s GIT [41,42]. In this way, avian hosts constitute a natural reservoir for *Campylobacter* spp., namely *C. jejuni* and *C. coli* [41]. According to the European Food Safety Authority (EFSA) and European Centre for Disease Prevention and Control (ECDC), the highest prevalence of *Campylobacter* is observed in fresh meat from broilers (37.5%) [31]. Although carriers of *Campylobacter* spp., chickens generally do not exhibit clinical signs [41]. Antibiotics have a limited role in the elimination of *Campylobacter* spp. by these animals, due to its high occurrence and commensal character in avian species, and can promote the emergence of resistant strains; therefore, biosecurity practices are the most important method for reducing *Campylobacter* infection at the production level [43].

Human infections are usually associated with the handling, preparation and consumption of contaminated poultry products, and occupational transmission has also been observed [39]. In humans, these pathogens cause gastroenteritis associated with diarrhea, abdominal pain, fever, nausea and vomiting, which usually occur between two and five days after infection. Symptoms are often mild and self-limiting. Antibiotic treatment is not usually required, but severe cases may be treated with macrolides, such as clarithromycin, azithromycin and erythromycin. Ciprofloxacin is not currently used, as resistance to quinolones is now considered to be too high for these antibiotics to be used as an empirical treatment [33,39,41,44]. Studies on the antimicrobial drug resistance profile of *Campylobacter* spp. isolated from broilers, laying hens, chicken carcasses and chicken meat revealed high frequencies of resistance to nalidixic acid, ampicillin, cephalexin, ciprofloxacin, erythromycin, gentamicin and tetracycline [30].

### 3.3. Escherichia coli

*E. coli* is a gram-negative bacillus belonging to the Enterobacteriaceae family [45]. It is an important bacterial species in the human–animal–environment triad, since it is a commensal inhabitant of the digestive tract of animals, including birds, being widely disseminated via fecal material [46]. This species is often studied as a marker of antimicrobial drug resistance, mainly due to its widespread distribution and capacity to harbor several genes in mobile genetic elements, serving as a source of antimicrobial drug resistance determinants to other bacteria [47].

Most *E. coli* are nonpathogenic; however, certain pathogenic serotypes may induce disease. There are several *E. coli* pathotypes, which can be divided into extraintestinal *E. coli* (ExPEC) and diarrhoeagenic *E. coli* (DEC). Avian pathogenic *E. coli* (APEC), an ExPEC, may induce colibacillosis in domestic birds, a disease characterized as a local or systemic syndrome that can be transmitted by oral or vertical routes or through inhalation. *E. coli*-associated infections are widely distributed among poultry of all ages. Birds can be asymptomatic until sudden death or present various forms of disease, such as septicemia, coligranuloma (Hjarre’s disease), air sac disease (chronic respiratory disease), swollen-head syndrome, venereal colibacillosis, cellulitis, peritonitis, salpingitis, orchitis, osteomyelitis/synovitis, panophthalmitis, omphalitis/yolk sac infection and enteritis [48]. Colibacillosis constitutes the most frequent infectious bacterial disease found in poultry, being responsible for significant economic losses due to the loss of productivity, increased mortality and condemnations of carcasses [29,48].

**Figure 4:**
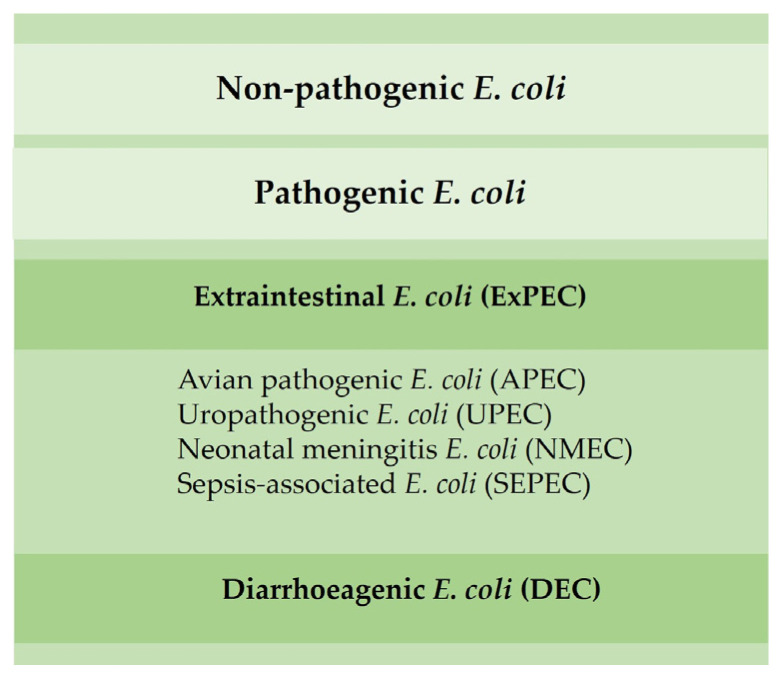
Main *E. coli* pathotypes found in poultry.

Other ExPEC pathotypes, such as uropathogenic (UPEC), neonatal meningitis (NMEC) and sepsis-associated *E. coli* (SEPEC), have already been identified in poultry and can promote disease in humans (Figure 4) [49]. *E. coli* isolated from poultry may be resistant to aminoglycosides, β-lactam groups (penicillins and cephalosporins) and fluoroquinolones [30].

### 3.4. Enterococcus *spp.*

*Enterococcus* species are ubiquitous and are commensals of the gastrointestinal microbiota of both humans and animals [50]. Some enterococcal strains have been used as probiotics [51], while others are known to be pathogenic, including in birds [52].

The transmission of enterococci can occur via vertical and horizontal routes. *E. cecorum* and *E. faecalis* are the most important species associated with avian disease. Pathogenic strains of *E. cecorum* have been associated with free thoracic vertebra (FTV) osteomyelitis in broilers [53], resulting in the paralysis of the posterior limbs, and with septicemia related to pericarditis or hepatitis, which can lead to death. In turn, *E. faecalis* can cause omphalitis and yolk sacculitis, which can lead to sepsis and the death of chicks in the first week of life. Surviving animals may develop chronic diseases, such as valve endocarditis, which can also lead to death [30].

*Enterococcus* spp. can easily acquire resistance determinants and, therefore, play a central role in AMR dissemination. Vancomycin-resistant *Enterococcus* (VRE) has been associated with economic losses in animal production and healthcare and associated with infections in humans [54]. Humans are exposed to enterococci from a variety of sources, including other humans, the environment and foods contaminated with animal’s intestinal microbiota. Certain species, such as *E. faecalis* and *E. faecium*, are a prominent cause of opportunistic infections in hospitalized humans, causing mild to fatal diseases, such as endocarditis, urinary tract infections or septicemia [50,52]. Studies previously performed have identified high levels of resistance against aminoglycosides (streptomycin), tetracyclines (doxycycline and tetracycline) and quinolones (ciprofloxacin and enrofloxacin) in enterococci isolated from poultry [30]. Vancomycin resistance, which is reported as infrequent, can be higher in isolates from chickens affected with FTV.

### 3.5. Methicillin-Resistant Staphylococcus aureus

*S. aureus* is considered the most common and pathogenic staphylococcal species isolated from poultry. Staphylococci are natural inhabitants of the skin and mucous membranes of healthy birds, being ubiquitous in the poultry environment [29,30]. The presence of *S. aureus* that is resistant to antimicrobials in production animals is a global health concern affecting both humans and animals [55]. Staphylococcal infections caused by *S. aureus* are a worldwide problem in poultry production, causing economic losses due to decreased production, increased mortality and the condemnation of carcasses. Infections caused by *S. aureus* include arthritis, synovitis, chondronecrosis, osteomyelitis, gangrenous dermatitis, subdermal abscesses (bumblefoot) and septicemia [29,30]. Moreover, some enterotoxin-producing strains can cause food poisoning in humans. Poultry-associated food poisoning can occur due to the contamination of carcasses with *S. aureus* at the processing phase, especially with enterotoxin-producing strains [56]. Regarding the presence of *S. aureus* in poultry, the major concern is the emergence of MRSA strains. Although they are infrequently isolated from poultry, MRSA can still be transmitted to humans through direct contact or through meat consumption [57]. Staphylococci from poultry can be resistant to amoxicillin, amoxicillin-clavulanic acid, ampicillin, cefoxitin, kanamycin, penicillin and tetracycline [30].

## 4. Strategies to Reduce Antimicrobial Drug Resistance in Poultry Production

Since the consumption of poultry meat is growing, the high density of animals in production flocks increases the risk of the transmission of infectious agents, including AMR bacteria. This prompts the need to find alternatives to replace or complement antibiotic usage in those settings and to evolve to a “post-antibiotic era” [58].

As previously described, there are several pathogens that are difficult to eliminate from poultry flocks, poultry meat and egg products, requiring improvements in all phases of the poultry production system. In the production phase, the optimization of cleaning procedures, improvement of biosecurity and implementation of adequate hazard analysis and critical control points are fundamental. At the retail level, it is crucial to take action on food handling and worker training, together with consumers’ education, to improve food safety awareness. Collectively, these actions offer opportunities to limit foodborne pathogen dissemination and reduce the risk of exposure to susceptible individuals; however, these measures may still be insufficient to protect humans from foodborne pathogens [33].

Interventions in poultry production can be grouped into two categories: pre-harvest and post-harvest interventions. At pre-harvest, measures to ensure animal health are applied primarily to prevent colonization and broiler infection by foodborne pathogens, via, for example, the administration of compounds in feed or drinking water. At post-harvest, measures applied aim to reduce or eliminate pathogens on carcasses or egg products. These measures focus on direct application on food, food packaging, surfaces and food processing equipment with the goal of minimizing the colonization or multiplication of pathogens and the spoilage of microorganisms during storage or retail [33,59].

Despite the availability and research on new substances, investigations usually focus on new methods to be applied at the flock production level, rather than on postharvest operations. This approach can be beneficial for two reasons. First, the ban of AGPs from poultry production led to the emergence of a market opportunity for alternative feed compounds showing health and performance benefits. Second, and from an overall food safety perspective, although reducing foodborne pathogens in processing plants is important, the focus should be on the live bird sector in order to reduce the pathogen loads before they enter the processing plants. However, the administration of alternative antimicrobial compounds to live birds through feed amendments has proven to be more challenging than anticipated [33]. In this sense, this review will focus on the pre-harvest application of nonconventional antimicrobial compounds, approaching, with greater depth, the reduction and eradication of pathogens at the flock level for the improvement of the flock’s health.

### 4.1. Husbandry

As previously described, one of the main objectives of the global action proposed by the WHO in 2015 is the reduction in infection incidence via effective sanitation and the application of hygiene and infection preventive measures [13]. The poultry industry needs to control infectious diseases, primarily through good husbandry and good farm management. If this point is addressed, all the other measures directed to pathogens and disease control can consequently be reduced [17]. This is valid for every step of the poultry production system from farm to fork. For example, when birds are transported to the processing facilities, if stressed, they excrete loose feces, which contaminate the bird itself and its surroundings, contributing to cross-contamination [33]. This highlights the importance of good transportation conditions. Another example is the importance of controlling flock density. Rawson, Dawkins and Bonsall, 2019 [60] concluded that high flock density is an essential factor responsible for *Campylobacter* spp. high microbial counts. *Campylobacter* becomes well established in large commercial flocks and becomes difficult to eradicate [33].

Hence, it is essential to develop innovative hygienic and management practices (focusing, for example, on housing and feeding systems) aiming to reduce, or even stop, the use of antimicrobials; develop and identify alternatives to antimicrobials, such as vaccines and supplements; and educate farmers and veterinarians to be in favor of a conscious attitude towards the importance of husbandry and the application of good practices [11].

### 4.2. Non-Conventional Antimicrobial Compounds

Due to the emergence of resistant microorganisms, research has focused on finding strategies alternative to conventional antibiotics, such as antimicrobial peptides, bacteriophages, probiotics and nanoparticles, as well as the use of alternative treatments. These solutions are considered fundamental to combating the dissemination of resistant microorganisms [17].

#### 4.2.1. Antimicrobial Peptides

Antimicrobial peptides (AMPs) are small proteins that can be found in almost every living organism. They evolved as a host defense mechanism against microorganisms and are important to innate immunity [17,61]. Generally, AMPs are small molecules (<10 kDa) with 12–50 amino acids, presenting broad-spectrum antimicrobial activity against bacteria, fungi, protozoa and viruses [62,63]. AMPs have several advantages: they can present several modes of action, are easily degraded in nature, present reduced accumulation, contribute to the enhancement of host immunity, have the ability to neutralize the activity of many microorganisms and seem to present a low resistance frequency [17,64]. The majority of AMPs act by disrupting the bacterial membrane via several mechanisms, including electroporation, non-lytic membrane depolarization, membrane destabilization, pore formation, membrane thinning or thickening, and oxidized lipid targeting [65]. However, some AMPs can also interact with intracellular targets, by inhibiting the cell wall, protein and acid nucleic synthesis, and by interfering with the bacterial metabolic turnover [66].

The healthy functioning of poultry’s GITs depends on the homeostasis between physical, chemical, microbiological and immunological components [5]. The lymphoid tissue present in the GIT (gut associated lymphoid tissue (GALT)) is responsible for the interaction with antigens and the establishment of an immune response [45]. The interactions between intestinal components, such as mucosa and glycocalyx, where AMPs can be naturally found, lead to the maturation of the GIT immune system [5]. These AMPs that naturally occur in the GIT of poultry can serve as a template for new antimicrobials, and rapid advances in peptide synthesis technology make the future prospect of the industrial application of synthetic AMPs in poultry promising [5,45]. Due to their bacteriostatic or bactericidal effect, AMPs may be used in the prophylactic control, or for the treatment, of bacterial infections, while simultaneously promoting broiler’s growth. AMPs have been shown to significantly reduce pathogenic bacterial loads within the avian gut, while increasing the population of beneficial bacteria and modulating intestinal microbiota [17,45]. The use of synthetic and recombinant AMPs as feed additives may result in benefits for poultry production by promoting immunomodulation, controlling potential pathogen outbreaks, reducing the development of AMR and decreasing the risk of antibiotic-resistant foodborne pathogen consumption by humans [5]. In some situations, various functions can be attributed to the same AMP. Table 1 gathers information on previous studies regarding the use of AMPs in poultry production. In addition, AMPs can act synergistically with conventional antimicrobials, allowing the improvement of the antimicrobial activity of these compounds [14].

The GIT encompasses various organs responsible for digestion and immunity. The microbiota of the GIT is related to the growth performance of animals since the presence of specific microorganisms may promote the absorption of nutrients. Moreover, by reducing the presence of pathogenic species and altering the microbiota, AMPs may help decrease the frequency and lethality of some infections [67]. Additionally, AMPs can contribute to improving animals’ growth by reducing the competition for nutrients in the small intestine, the production of growth-depressing metabolites and the production of intestinal proinflammatory factors, which, in turn, can enhance poultry production parameters and feed intake [68].

Several diseases affect livestock production by causing intestinal mucosa inflammation and diarrhea associated with morphological changes in the intestinal epithelium [69]. AMP utilization has been demonstrated to contribute to health recovery by stabilizing the epithelial barrier integrity and boosting intestinal epithelium colonization. Furthermore, some AMPs can act by inhibiting pro-inflammatory cytokine production or modulating dendritic and T-cell response [17].

##### AMP Use as Growth Promoters

Several studies have already been carried out to determine the potential activity of AMPs as growth promoters [5]. Microcin J25 (MccJ25), a bacteriocin produced by a fecal *E. coli* with strong inhibition ability against other *E. coli* and *Salmonella*, has been shown to promote growth performance, influence intestinal microbiota and improve intestinal health [70,71]. Nisin, a bacteriocin produced by *Lactococcus lactis*, is currently being used in the food industry as an additive (GRAS, E234 in EU) due to its inhibitory capacity against putative bacterial pathogens. Although some studies have been performed aiming at its use in poultry [72,73,74], the EFSA has not approved its utilization in those settings. As such, nisin for use in livestock diets is still forbidden, including in poultry, and is not registered as a feed additive [72]. Various studies have demonstrated that the inclusion of nisin in feed changes the animal’s GIT microbiota, reducing potential pathogens in the ileum, such as the *Bacteroides*-*Prevotella* cluster, *Enterobacteriaceae* [73] and *Clostridium perfringens* [74]. This culminates in a reduction in pathogens in the GIT, lowering the competition for nutrients between the bacteria and the host, and, thus, improving energy utilization [72]. AS7, a bacteriocin produced by *Carnobacterium divergens* and investigated as a feed supplement, was demonstrated to have a positive role in growth promotion and antibacterial effects in broiler chickens [75]. The addition of Cecropin A-D-Asn, a recombinant AMP, to broilers’ feed, was shown to inhibit gut bacterial growth, improve nutrient utilization and intestine structure, and promote broiler growth [76]. Furthermore, the use of the synthetic AMPs A3 and P5 was able to increase the growth performance of broilers, with additional benefits such as increased nutrient uptake and reduced intestinal damage [77,78].

##### AMP Use as Immunomodulators

Other studies have reported a similar effect of several AMPs, derived from different sources, to reduce bacterial infections in broiler flocks through immune modulation [78,79]. AMPs can modulate the intestinal expression of proinflammatory cytokines, such as IL-2 and IL-6, and of anti-proinflammatory molecules, such as IL-10 [45,80]. Moreover, AMP supplementation improves intestinal morphometric parameters, including villus height and villus surface area, and productive parameters, such as feed conversion ratio [45]. Notably, supplementation of feed with exogenous AMPs mimics the physiological release of endogenous AMP, such as cathelicidin-B1 [80]. Therefore, AMPs can be considered major alternatives to maintaining intestinal balance in avian species [5]. Kogut, Genovese, He, Swaggerty and Jiang, 2013 [81] studied the application of a group of small cationic peptides (BT) with known immune modulatory properties produced by a Gram-positive bacterial species from soil, *Brevibacillus texasporus*, to broiler chickens. The authors demonstrated that these AMPs may be useful alternatives to antibiotics, acting as local immune modulators in neonatal poultry and providing prophylactic protection against *Salmonella* infections. A study by Aguirre et al., 2015 [82] demonstrated that bovine lactoferrin (bLf) had beneficial effects in broiler chickens, promoting the improvement of body weight and feed conversion and the enhancement of intestinal morphology. Liu et al., 2008 [83] determined the effect of the oral administration of rabbit *Sacculus rotundus* antimicrobial peptides (RSRP) on the intestinal mucosal immune responses in broilers. The results indicated that the presence of RSRP improved the structure of the intestine and stimulated intestinal mucosal immunity during growth. Ma et al., 2020 [84] studied the effects of diet supplementation with recombinant plectasin in broilers, showing its beneficial effects on growth performance, intestinal health and innate immunity.

##### AMPs Used for Infection Therapy in Poultry

Finally, AMPs also present an enormous potential in controlling poultry infectious diseases [85].

***Salmonella***

Cathelicidins were shown to present relevant antimicrobial activity against *Salmonella*. For example, synthetic human cathelicidin LL-37, released by neutrophils, has demonstrated strong antimicrobial activity against different *Salmonella* strains and immunomodulation effects [86]. Cathelicidin BF is one of the most potent cathelicidins known. Its administration in mice reduced infection by *Salmonella* resistant to streptomycin, accentuating the potential of AMPs in eliminating antibiotic resistant bacteria. Another cathelicidin, CATH-2, which is derived from chickens, showed *in vitro* antibacterial activity against *S.* Enteritidis and *S.* Typhimurium [87]. An *in vivo* study performed by Roque-Borda et al., 2021 [64] evaluated the effect of a peptide derived from the skin of the amphibian *Hypsiboas albopunctatus*, Ctx(Ile^21^)-Ha, known for its high antimicrobial activity against some important public health pathogens in laying hens. The authors concluded that after a challenge with *S.* Enteriditis and AMP treatment there was a reduction in younger chicks’ mortality in the first days of life. However, the investigation of the potential of avian AMP to reduce *Salmonella*’s infections in poultry is still ongoing.

***E. coli***

An *in vivo* study performed by Daneshmand et al., 2019 [45] demonstrated that the use of cLF36, a lactoferrin-derived peptide, is capable of decreasing infection in broilers challenged with enterotoxigenic *E. coli* by modulating the expression of cytokines IL-2 and IL-6 and mucine. The addition of cLF36 to feed reduced the population of *E. coli* and *Clostridium* spp. by 25% and 20%, respectively. Additionally, the number of *Lactobacillus* spp. and *Bifidobacterium* spp., species beneficial to poultry’s GIT microbiota, increased up to 36% [45]. Another study has also evaluated the application of a recombinant peptide derived from camel milk, cLFchimera, which presented a strong antimicrobial effect against two different *E. coli* strains in birds [88].

***Campylobacter* spp.**

An *in vitro* study performed by Line et al., 2022 [61] showed that six AMPs, namely, NRC-13 Pleurocidin, RL-37, Temporin L, Cecropin A-Magainin 2 hybrid, Dermaseptin and C_12_K-2β_12_, had the capacity to inhibit *Campylobacter* growth. One of these AMPs, C_12_K-2β_12_, is heat- and acid-stable, making it an attractive compound for *in vivo* delivery to poultry. Moreover, another *in vitro* study showed that three purified bacteriocins produced by *Paenibacillus polymyxa* and one from *Bacillus circulans* NRRL B-30644 presented antagonistic activity against *Campylobacter* from broiler chickens [89]. Nevertheless, it is important to note that further *in vivo* studies, aiming at evaluating the inhibition of *Campylobacter* spp. infections in poultry, must be conducted.

***C. perfringens***

Pediocin A, a bacteriocin produced by *Pediococcus pentaceus* FBB61 [90], has demonstrated antimicrobial activity against Gram-positive bacteria, such as *C. perfringens* type A. Pediocin A was administered via feed to broilers infected with *C. perfringens* type A, which resulted in the increased growth of the animals [85,91]. In another study, Sublacin, a peptide produced by *Bacillus subtilis* 168, was administered to poultry via drinking water. Authors demonstrated that this AMP could be used as a potential antimicrobial agent to control necrotic enteritis caused by *C. perfringens* without causing changes in the *Lactobacillus* community [92].

**Table 1:** *In vitro* and *in vivo* studies evaluating AMPs activity against poultry-associated foodborne pathogens (nd—not determined; na—not applicable).

AMP	Origin	Target (Gram)	Trial	Dosage	Effect	Study
3 bacteriocins	*Paenibacillus polymyxa* *B. circulans*	*Campylobacter* (−)	*In vitro*	na	Antimicrobial activity	[89]
Gallinacin-6	*Gallus gallus domesticus*	*C. jejuni* (−), *S. enterica* (−)*, C. perfringens* (+), *E. coli* (−)	*In vitro*	na	Antimicrobial activity	[93]
RSRP	*Oryctolagus cuniculus—sacculus rotundus*	*E. coli* (−)	*In vivo*	0.1 mg of RSRP on d 7, 14, 21 and 28	Immunomodulation; alteration of intestinal morphology	[83]
AS7	*Carnobacterium divergens* AS7	*C. perfringens* (+)	*In vivo*	200 AU/g of feed for 42 days	Improvement of growth performance; alteration of intestinal microbiota	[94]
A-D-Asn	*Pichia pastoris*	-	*In vivo*	Basal diets with a A-D-Asn supplementation at 0, 2, 4, 6 and 8 mL/kg	Improvement of growth performance	[76]
BT	*Brevibacillus texasporus*	*S.* Enteritidis (−)	*In vivo*	24 ppm BT peptide-supplemented diet	Immunomodulation in neonatal poultry; antimicrobial activity (prophylactic protection against *Salmonella* infections)	[81]
Nisin	*Lactococcus lactis* subsp. *lactis*	-	*In vivo*	Diet supplemented with various concentrations of nisin (100, 300, 900 and 2700 IU/g)	Improvement of growth performance; modulation of the GIT microbial ecology	[73]
CATH-2	Chicken	*S.* Enteritidis (−) *S.* Typhimurium (−)	*In vitro*	na	Antimicrobial activity	[87]
A3	Analog of *Helicobacter pylori* 2–20	-	*In vivo*	Basal diet supplemented with 60 or 90 mg/kg AMP-A3	Improvement of growth performance; alteration of intestinal microbiota	[77]
P5	Analog of hybrid AMP CA-MA	-	*In vivo*	Basal diet supplemented with 40 and 60 mg/kg AMP-P5	Improvement of growth performance; alteration of intestinal microbiota	[78]
Sublacin	*B. subtilis* 168	*C. perfringens* (+)	*In vivo*	Chickens supplemented with sublancin at 2.88, 5.76 or 11.52 mg activity/L of water	Antimicrobial activity; alteration of intestinal morphology	[92]
Lactoferrin (bLf)	*Bos taurus*	*E. coli* (−) *Salmonella* (−)	*In vivo*	Diets with 130, 260 and 520 mg bLf/kg feed during the starter stage	Alteration of intestinal morphology	[82]
Nisin	*Lactococcus lactis* subsp. *lactis*	-	*In vivo*	35-day administration of nisin at 2700 IU kg^−1^ through diet	Improvement of growth performance	[74]
Microcin J25 (Mcc)	Fecal strain of *E. coli*	*E. coli* AZ1 (−) *Salmonella* CVCC519(−)	*In vivo*	Basal diet with 0, 5 and 1 mg/kg Mcc J25	Decrease in intestinal inflammation; alteration of intestinal microbiota; improvement of growth performance	[70]
Nisin	*Lactococcus lactis* subsp. *lactis*	-	*In vivo*	Administration of nisin in 2700 IU/kg via diet	Improvement of growth performance	[95]
cLFcuimera	Camel lactoferrin-derived peptide	*E. coli* (−)*, S.* Enteritidis (−), *S. aureus* (+)	*In vitro*	na	Antimicrobial activity on both Gram-positive and Gram-negative avian pathogenic bacteria	[88]
cLF36	Camel lactoferrin-derived peptide	*E. coli* (−) *Clostridium* spp. (+)	*In vivo*	20 mg/kg AMP	Improvement of growth performance; immune modulator; alteration of intestinal microbiota	[45]
Plectasin (Ple)	*Pseudoplectania nigrella*	-	*In vivo*	Basal diet supplemented with 100 and 200 mg Ple/kg	Immunomodulation	[84]
Ctx(Ile^21^)-Ha	*Hypsiboas albopunctatus*	*S.* Enteriditis (−)	*In vivo*	20 and 40 mg of Ctx(Ile21)-Ha/kg were included in the diet for 28 days	Antimicrobial activity	[64]
11 AMPs	Chemically synthesized	*C. jejuni* (−)	*In vitro*	na	Antimicrobial activity	[61]

#### 4.2.2. Bacteriophages

There has been an increased interest in research regarding the application of bacteriophages in the poultry industry [29]. Bacteriophages (or phages) are viruses that specifically target and infect bacteria. Phages were discovered in 1915 [96], but research focusing on their usage as antimicrobials decreased with the spread of antibiotic use [16].

Phages are globally ubiquitarian in the environment, being found in all habitats, including in water, plants and food, and are relatively easy to isolate. They are frequently consumed by humans (via food or water) and are considered non-pathogenic. Moreover, phages have been recognized as important components of the human microbiome, being prevalent in the human gut virome [97]. Bacteriophages may be present in high concentrations in the farm environment, but only a small percentage may have specific action against a target pathogen [98]. The best source of phages is the environment where the host bacterium is prevalent [99,100].

Phages are obligate bacterial parasites, using the prokaryotic cell to replicate. Depending on their interactions with bacteria and their life cycle, phages can be divided into two types: lytic (or virulent) and lysogenic. During the lytic cycle, a bacteriophage infects a target bacterium, replicates, kills the bacterium through lysis, and releases multiple progeny phages. These progeny phages can infect other bacterial cells, thereby repeating the cycle [101]. Lytic phages have several potential applications. In contrast, the lysogenic cycle does not result in the lysis of the host cell, nor in progeny production. Instead, it leads to the integration of phage genetic material into the bacterial genome, and its transmission into new cells upon cell division. Finally, certain bacteriophages have the ability to perform both lytic and lysogenic cycles, depending on environmental triggers [102].

Bacteriophages are classified into many orders and 15 families. The vast majority of phages (96%) belong to the order Caudovirales, which correspond to phages with tails. This order is divided into three families: Siphoviridae (including 61% of tailed phages), Myoviridae (25%), and Podoviridae (14%) [103]. Phages possess very high specificity for one (monovalent phage) or similar (polyvalent phage) bacterial species. On one hand, the selective ability of phages to attack certain bacteria allows for the elimination of specific microorganisms; on the other hand, it restricts their use for broad therapeutic purposes [29]. Phage specificity towards target bacteria is dependent on cell surface receptors, such as outer membrane and lipopolysaccharide proteins and flagella components [104].

In addition to therapies using only bacteriophages, it is described that phages can be more effective when applied in combination with conventional antibiotics. This phenomenon is known as phage–antibiotic synergy. These combinations allow the application of antibiotics in sub-inhibitory doses, leading to a reduction in the harmful effects of antibiotics in animals and humans. In addition, the combination of phages with antibiotics can potentiate antibiotic function, prolonging or restoring their activity against bacteria [105,106]. To the authors’ best knowledge, this synergism is not fully characterized regarding the application of phages in poultry production; however, it is a factor that should be considered in the development of protocols for the treatment of bacterial infections using bacteriophages.

Several studies have addressed the efficacy of phages in reducing bacterial counts or controlling bacterial infections in poultry. Phages can be applied in the poultry industry with three different aims: food biocontrol, disinfection (post-harvest) and phage therapy directed to infections (pre-harvest) [29].

Regarding food biocontrol, phages are used to reduce food contamination, promoting the removal and neutralization (inactivation) of microorganisms in food products and delaying putrefaction. Specifically, phages may be used as food biopreservatives, via the direct application on food or as food packaging, including on raw meat and ready-to eat (RTE) products. In fact, there are some phages being commercialized as biopreservation agent products acting against *Listeria monocytogenes*, *Salmonella*, *Shigella* and *E. coli* [29,59].

For disinfection, phages can be applied to the decontamination of food-contact surfaces, equipment and the skin of poultry carcasses, with the aim of reducing bacterial loads [59].

##### Phage-Mediated Control at Pre-Harvest

Bacteriophages function in more specific ways compared to antibiotics; however, only lytic bacteriophages are suitable for phage therapy. It should be noted that antibiotic treatment not only kills the pathogenic bacteria but also affects the normal intestinal microbiota, potentially leading to dysbiosis, immunosuppression and secondary infections [107]. In this way, bacteriophage treatment represents an excellent tool for the treatment of bacterial infections in poultry. Table 2 shows the results from studies conducted about the use of bacteriophages against pathogens in poultry production.

***Salmonella***

Since *Salmonella* is one of the most important foodborne pathogens, the search for bacteriophages directed towards this agent is of major importance. In 1991, an experiment was conducted on newly hatched chickens challenged with *S.* Typhimurium [108]. After bacterial challenging and phage inoculation, a reduction in the microbial load was observed in some portions of the GIT. Bardina, Spricigo, Cortés and Llagostera, 2012 [109] concluded that bacteriophage cocktails composed of various phages are more effective in promoting *Salmonella* inhibition than the administration of just one phage, promoting a significant reduction in *Salmonella* counts in chicken cecum after repeated treatment. Hong et al., 2013 [110] showed that bacteriophages may be effective alternatives to antibiotics for the control of fowl typhoid disease caused by *S.* Gallinarum in layer chickens. In this study, chickens were fed with bacteriophages for 7 days before a bacterial challenge and for 21 days after the challenge, and mortality rates significantly decreased in the challenged chickens treated with bacteriophages. Nabil, Tawakol and Hassan, 2018 [111] reported the presence of *Salmonella*-specific bacteriophages in sewage samples from a poultry farm. Those bacteriophages were administrated to chicks before oral *Salmonella* challenge, and were subsequently followed by four successive phage treatments. At the end of the trial (after the fifth and last dose), no bacteria were detected in the cecum, indicating that *Salmonella* was eliminated by phage treatment.

The world’s first commercially available bacteriophage product for application in poultry production is the Biotector S1^®^ (CJ Cheil Jedang Research Institute of Biotechnology, Seoul, Republic of Korea), which can be used as a feed additive to control *S.* Pullorum and *S.* Gallinarum in poultry. In a study using this product, the mortality rate in chickens challenged with *Salmonella* and receiving Biotector S1^®^ decreased and performance was improved in the phage treated group [59]. Another phage cocktail available, SalmoFREE^®^(Theseo, Laval, France), is considered safe for administration via drinking water, which does not affect animals’ production parameters. This product can reduce *Salmonella* levels in cloaca to 0% after a 33-day treatment [112]. Bafasal^®^ (Proteon Pharmaceuticals, Poland), another phage-based product, can also be administrated via drinking water to birds and has the ability to reduce *Salmonella* levels, improve feed conversion rates and reduce mortality. In addition, it has a prophylactic and a post-infection effect and its administration does not require a waiting period for meat and eggs [113,114].

***Campylobacter* spp.**

Most of the phages exhibiting specificity for *Campylobacter* belong to the family Myoviridae, and to a lesser extent, to Siphoviridae [115]. *Campylobacter* phages have been isolated from retail poultry, the feces and intestines of chickens and ducks, abattoir effluents, sewage, human feces and poultry manure [99]. There are already some *in vivo* studies on the use of phage treatment for the reduction in *Campylobacter* GIT colonization and infection in poultry. In 2005, Wagenaar, Bergen, Mueller, Wassenaar and Carlton [116] compared the efficacy of a single *Campylobacter* phage with a cocktail of two-phages, administered to live broilers, concluding that phage-treatment can decrease *C. jejuni* colonization in broiler caeca when phages are used in a preventive way, as well as therapeutically, and that a cocktail of two phages can reduce the rate of phage-resistant mutant development, when compared to single phage usage. Loc Carrillo et al., 2005 [117] studied the effectiveness of two phages towards *Campylobacter* strains, demonstrating that the best reduction in bacterial load was achieved after 24–48 h, and that this decrease depended on phage amount and time of administration; however, substantial differences were identified between *in vitro* and *in vivo* results, which highlights the importance of developing *in vivo* studies. In 2009, El-Shibiny et al. [118] evaluated the application of the CP220 phage to broilers colonized with *C. coli* or *C. jejuni*, concluding that only high doses of phages were able to reduce *C. coli* caecal counts within 48 h, while a more extensive reduction in *C. jejuni* HPC5 levels occurred at 24 h post-administration. In 2010, Carvalho et al. [119] tested the efficacy of a phage cocktail composed of three phages for the control of *Campylobacter* infections in poultry and also evaluated the effectiveness of two administration routes (oral gavage and feed supplementation). The authors observed that the cocktail targeted both *C. jejuni* and *C. coli* and that administration via feed led to an early and more sustainable reduction in *Campylobacter* compared to administration by oral gavage. Kittler, Fischer, Abdulmawjood, Glünder and Kleina, 2013 [120] tested a phage cocktail composed of four group III phages in three commercial broiler farms. In this study, one of the experimental groups showed a significant reduction in *C. jejuni*, with a maximum reduction being achieved 1–4 days prior to slaughter. The fact that sometimes genotypically identical *C. jejuni* strains within flocks exhibit different phage susceptibility profiles was another important conclusion from this study, linking the lack of efficacy with the genotypic variability of *C. jejuni* isolates. In another study, Fischer, Kittler, Klein and Glünder, 2013 [121] tested a phage cocktail, as well as a single phage, observing a significant reduction in *Campylobacter* after one to four weeks of treatment and also concluding that phage cocktail administration delayed the emergence of phage resistance.

Currently, to the authors’ knowledge, there are no commercially available phage-based products directed to *Campylobacter*. As previously mentioned, there are several studies being performed on *Campylobacter* phage treatment; however, since multiple genotypes of *Campylobacter* can be present in a flock, this genetic diversity can contribute to the decreased effectiveness of phage therapy and resistance development.

***E. coli***

In 1998, Barrow, Lovell and Berchieru [122] studied the efficacy of a bacteriophage isolated from human sewage, bacteriophage R, in preventing and treating septicemia, cerebritis and meningitis in chickens inoculated intramuscularly or intracranially with *E. coli*. The results showed that phages could reach the animals’ brain and that animals treated with phages before the *E. coli* challenge did not develop disease, which may indicate that phages can persist long enough in the tissues to allow for their application as a prophylactic measure for colibacillosis, as well as for infection treatment. Huff, Huff, Rath, Balog and Donoghue, 2003 [123] tested the application of a two-bacteriophage mixture as an aerosol spray and intramuscular injection treatment to birds immediately after challenging the animals with *E. coli*, observing that this method provided significant protection to the birds and decreased mortality from 50 to 20%. However, the bacteriophage aerosol spray was not effective when applied 24 or 48 h after the birds were challenged with *E. coli*. In fact, treating this severe respiratory *E. coli* infection with a single injection into the thigh muscle was found to be much more effective, significantly reducing mortality when administered immediately after the *E. coli* challenge, as well as at 24 and 48 h after the challenge. In 2009, the same authors [124] demonstrated that bacteriophage administration via an aerosol spray to seven-day-old chicks prior to a challenge with *E. coli* could prevent airsacculitis caused by this bacterial agent. Moreover, the effectiveness of treatment with bacteriophage seems to be dependent on the circulating bacteriophage titers. After the administration of phages via an aerosol spray, only a few animals had detectable phage levels in the blood, in contrast to the animals subjected to intramuscular injection.

Other authors compared the efficacy of an antibiotic (chloramphenicol) and oral phage therapy (phage Esc-A, isolated from sewage) against enteropathogenic *E. coli* in 20-day-old chickens [125]. In the second week of treatment, the birds receiving phages presented no diarrhea, while 12.4% of the birds receiving the antibiotic presented diarrhea, along with 25.2% of those in the control group (treated with water). The death rate was 14.8% in the control group, which was two and five times higher than in the antibiotic and phage groups, respectively. In addition, oral phage therapy caused no secondary effects in the chickens when compared with antibiotic treatments. This can be explained by the fact that the phage treatment has a higher specificity and does not affect beneficial bacteria present in the gut, which is very important for maintaining intestinal homeostasis.

To our knowledge, phage-based products for colibacillosis treatment in poultry are still not available on the market; however, there are two substances available that can be applied topically to live animals for the reduction in *E. coli* loads, Ecolicide PX™ (Intralytix, Columbia, MD, USA) and Finalyse^®^ (Arm & Hammer Animal and Food Production, Princeton, NJ, USA) [59].

***Staphylococcus aureus***

Phages infecting *Staphylococcus aureus* belong to the Podoviridae, Siphoviridae and Myoviridae families. From the therapeutic point of view, Myoviruses are considered the most promissing phages against *S. aureus*. Although Podoviruses are strictly lytic, they rarely occur in nature. Despite showing strong specificity to *S. aureus*, these bacteriophages may carry enterotoxigenic genes, which make them pointless for phage therapy [126]. To our knowledge, there are no phage preparations commercially available for the prophylaxis, or the treatment, of *S. aureus* infections in poultry.

**Table 2:** *In vivo* studies evaluating bacteriophages activity against poultry-associated pathogens (nd—not determined).

Phage	Origin	Target (Gram)	Administration Route	Dosage (PFU—Plaque-Forming Unit)	Effect	Study
Phage type 14, type 40 and type 141	Raw human sewage	*S*. Typhimurium (−)	Feed	10^5^ and 10^10^ PFU/mL	Reduction in the viable numbers of *S.* Typhimurium in the crop, small intestine and caeca for up to 12 h after inoculation with smaller reductions in the liver at 24 and 48 h after infection	[108]
Phage R	Human sewage	*E. coli* (−)	Intramuscular Intracranial	10^2^–10^6^ PFU 10^6^–10^8^ PFU	Long persistence of phages in tissues, which may be useful in prophylaxis and treatment of colibacillosis	[122]
DAF6 and SPR02	Municipal and poultry processing waste	*E. coli* (−)	Aerosol Intramuscular	Aerosol spray: 7.65 × 10^8^ (DAF6) and 2.83 × 10^9^ (SPR02) PFU/mL; injection: 1.88 × 10^9^ (DAF6) and 6.35 × 10^8^ (SPR02)	Successful treatment of *E. coli* infection by intramuscular injection, which also could be used to prevent colibacillosis in poultry	[123]
Phage 69 (NCTC 12669) and Phage 71 (NCTC 12671)	National Collection of Type Cultures in the UK	*C. jejuni* (−)	Feed	4 × 10^9^ to 2 × 10^10^ PFU	Significant decrease in *Campylobacter* colonization	[116]
CP8 and CP34	Ceca and upper and lower intestines of chicken	*Campylobacter* (−)	Feed	log_10_ 5, 7 and 9 PFU	Decrease in the bacterial load depending on the amount of phage and time of administration	[117]
Esc-A	Sewage	*E. coli* (−)	Oral	10^5^ PFU	More efficient decrease in the death rate compared to chloramphenicol treatment	[125]
CP220	nd	*C. jejuni* (−) *C. coli* (−)	Oral gavage	log_10_ 5, 7 and 9 PFU	Decrease in *Campylobacter* colonization	[118]
phiCcoIBB35, phiCcoIBB37 and phiCcoIBB12	Poultry intestinal contents	*C. jejuni* (−) *C. coli* (−)	Oral gavage/feed	Phage cocktail with 1 × 10^6^–1.5 × 10^7^ PFU	Reduction in the number of *C. jejuni* (Experiment 1) and *C. coli* (Experiment 2) colonization in chickens	[119]
UAB_Phi20, UAB_Phi78 and UAB_Phi87	Chicken cloacae and pig rectal swabs	*Salmonella* spp. (−)	Oral	Phage cocktail with 10^10^ PFU/animal	The frequent treatment of the chickens with bacteriophages, especially prior to colonization of the intestinal tract by *Salmonella*, is required to achieve effective bacterial reduction over time	[109]
ST4, L13 and SG3	Sewage water treatment	*S.* Gallinarum (−)	Feed	Phage cocktail with 10^8^ PFU/kg	Significant decrease in bacterial isolation from the organs and mortality in chickens treated with the bacteriophages	[110]
Phages NCTC12672, 12673, 12674 and 12678	British phage typing scheme	*C. jejuni* (−)	Drinking water	Phage cocktail with log_10_ 5.8 to 7.5 PFU/bird	Decrease in *Campylobacter* load	[120]
Phages 1 (NCTC 12673), 2 (NCTC 12674), 5 (NCTC 12678) and 13 (NCTC 12672)	National Collection of Type Cultures	*C. jejuni* (−)	Oral	Single phage or a four-phage cocktail (10^7^ PFU/bird)	Permanent reduction in *Campylobacter* load by the phage cocktail, as well as by the single phage. However, the cocktail delayed the emergence of phage resistance	[121]
nd	Sewage water taken at broiler farm	*S.* Typhimurium (−) *S.* Enteritidis (−)	Oral	1.18 × 10^11^ PFU/chick to1.03 × 10^12^ PFU/chick	No detection of *Salmonella* in the cecum after the last (5th) dose	[111]

#### 4.2.3. Probiotics

The prohibition of antibiotics supplementation of poultry feed in the EU and other countries has led to a shift towards the use of new substances for the prophylactic control of some pathogens in broiler chickens at the farm level [127].

According to the WHO, probiotics are “live microorganisms which when administered in adequate amounts confer a health benefit on the host” [22,128]. A probiotic preparation must respect some requirements to be considered functional: probiotic bacteria should be resistant to the acidic pH of the environment; easily adhere to the intestinal epithelium; and maintain the intestinal microbiota at the appropriate physiological level [22,51]. Being live microorganisms, probiotics can stimulate gut microbiota, improving the host’s health [127]. These beneficial microbes can act via several mechanisms, including the maintenance of the normal intestinal microbiota, the competitive use of nutrients or sites of bacterial adhesion; metabolism change, by increasing digestive enzyme activity and decreasing bacterial enzyme activity and ammonia production; improvement of feed intake and digestion; and stimulation of the immune system [51,127]. Most of the effective probiotics are lactic acid bacteria (LAB), including those from the genera *Lactobacillus* and *Pediococcus*, which are normally found in the GIT of vertebrates and invertebrates [129]. Another type of probiotics, allochthonous probiotics, include microorganisms that are not usually found in the GIT, such as *Saccharomyces* and spore-forming *Bacillus* [129].

In broilers, probiotic species belonging to *Lactobacillus*, *Streptococcus*, *Bacillus*, *Bifidobacterium*, *Enterococcus*, *Aspergillus*, *Candida* and *Saccharomyces* have been shown to present a beneficial effect on performance, the modulation of intestinal microbiota and pathogen inhibition, intestinal histological changes, immunomodulation and on the microbiological meat quality, and have been used to integrate probiotic formulations. Contrarily to antibiotics, probiotics can be employed as growth promoters [51]. To our knowledge, thirty probiotic preparations are currently registered in the EU [22,51].

Lactic acid bacteria are known to contribute to a healthy intestinal environment, delivering enzymes and other beneficial substances to the GIT [51]. Several studies have already been performed aiming to evaluate LAB contribution to the normal microbiota of chickens [126,127,128,129,130]. Table 3 briefly presents several studies on the use of probiotics in poultry production.

The probiotic FloraMax-B11^®^ (Pacific Vet Group, Fayetteville, AR, USA), composed of 11 *Lactobacillus* strains (three of *L. bulgaricus*, three of *Limosilactobacillus fermentum* (ex *Lactobacillus fermentum* [131]), two of *Lacticaseibacillus casei* (ex *Lactobacillus casei* [131]), two of *Limosilactobacillus fermentum* (ex *Lactobacillus cellobiosus* [131]), and one of *L. helveticus*), was shown to successfully be able to reduce *Salmonella* spp. when applied via drinking water [129]. A study by Vicente et al., 2007 [132] also investigated the effect of the previously referred probiotic after administration via drinking water, observing that it significantly reduced mortality in poultry farms and increased animals’ performance. Another study evaluating FloraMax-B11^®^ in *Salmonella*-challenged broiler chickens also observed a reduction in bacterial loads in the GIT [133]. A study by Shivaramaiah et al., 2011 [134] also demonstrated that heat-resistant spore-forming *Bacillus* can markedly reduce *Salmonella* and *Clostridium* when administered in high loads. In fact, several studies have been performed to evaluate the effects of probiotics on *Salmonella* infections [133,134,135,136,137,138,139,140,141], and probiotics are already being used in the poultry industry for preventing or reducing *Salmonella* colonization orinfections, and enhancing growth performance in broiler chickens [127,134]. Moreover, several studies have already been performed that aimed at determining the potential role of probiotics in *Campylobacter* inhibition [142,143,144,145,146], and demonstrated that lactobacilli and bifidobacteria have the potential to inhibit *Campylobacter* spp. growth [147].

**Table 3:** *In vivo* studies evaluating effects of probiotics on poultry’s health (nd—not determined; na—not applicable).

Probiotic	Microorganisms	Target (Gram)	Dosage (CFU—Colony-Forming Unit)	Effect	Study
FM-B11^®^	*Lactobacillus* spp.	-	10^6^ CFU/mL via drinking water	Significant reduction in mortality and increase in broiler chick performance	[132]
FM-B11^®^	Eleven lactic acid bacterial isolates	*S.* Enteritidis (−)	Oral doses of 10^4^,10^6^ and 10^8^ CFU/bird	Possible reduction in *Salmonella* Enteritidis in neonatal chicks.	[133]
FM-B11^®^	*Lactobacillus* spp.	*S.* Enteritidis (−)	4 × 10^6^ CFU/mL via oral gavage	Significant reductions in the concentrations of *S.* Enteritidis within the ceca, and the timing of FM-B11^®^ treatment affects *S.* Enteritidis-associated reductions	[135]
-	*Bacillus* spp.	*S.* Typhimurium (−)	Directly fed microbials at 10^6^ spores/g of feed	Significantly lower cecal *S.* Typhimurium load and increased performance	[134]
Gallipro^®^	*B. subtilis*	*S.* Enteritidis (−)	0.02% probiotic of diet supplementation	No significant effect at non-contaminated environment, showing a greater efficacy at a pathogen contaminated environment and improving immune response of infected chickens	[136]
-	*E. faecium* PXN33 *Ligilactobacillus salivarius* (ex *Lactobacillus salivarius* [131]) 59	*S.* Enteritidis (−)	Oral gavage with 1 × 10^9^ CFU of probiotics	Prevention of *S.* Enteritidis colonization of poultry	[137]
-	*Bacillus* spp.	*S.* Typhimurium (−)	na	Reinstatement of the microbial genera displaced by *S.* Typhimurium challenge	[138]
-	*Bacillus* spp.	*S.* Enteritidis (−)	Feed supplemented in concentration of 454 g/ton	Reduction in the load of *Salmonella* in the ceca.	[139]
-	*B. subtilis* CSL2	*Salmonella* (−)	Feed supplemented in concentration of 1.0 × 10^7^ CFU/g of feed	Modulation in the microbiota, potentially protecting against *S*. Gallinarum infection	[140]
-	*L. salivarius*	*S.* Pullorum (−)	Feed supplemented in concentrations of 10^7^, 10^8^ and 10^9^ CFU/kg of feed	Enhancement in *S.* Pullorum infection resistance in broilers challenged with Aflatoxin B1	[141]
PrimaLac (Star Labs, St. Joseph, MO, USA)	*Lactobacillus acidophilus*, *Lactobacillus casei*, *Bifidobacterium thermophilus* and *Enterococcus faecium*	*C. jejuni* (−)	Minimum of 1.04 × 10^8^ CFU/g	Reduction in the presence of *C. jejuni*, but no significant effect on the growth performance of broilers	[142]
-	*Lactobacillus plantarum* PCS 20 and *Bifidobacterium longum* PCB 133	*C. jejuni* (−)	Oral gavages in concentration of 10^8^ CFU for 15 days	*B. longum* PCB 133 led to significant reduction in *C. jejuni* concentration in poultry feces.	[143]
-	*Lactobacillus gasseri* SBT2055	*C. jejuni* (−)	Oral gavages in concentration of 1 × 10^8^ CFU CFU for 14 days	Significant reduction in cecum colonization by *C. jejuni* at 14 days after infection.	[144]
-	*Lactobacillus gasseri* SBT2055	*C. jejuni* (−)	na	Significant reduction in cecum colonization by *C. jejuni*.	[145]
-	*Bacillus* spp. *L. salivarus* subsp. *salivarius* *L. salivarus* subsp. *salicinus*	*Campylobacter*(−)	na	These strains had significantly reduced *C. jejuni* counts at 14 days after infection.	[146]

#### 4.2.4. Nanoparticles

Nanotechnology is an innovative technology with various biomedical applications, and its implementation in the poultry industry has been studied [148]. Nanoparticles (NPs) have unique physical and chemical properties, which make them a target of attention due to their potential use in a range of diverse areas [149,150]. Based on their composition, NPs can be classified as inorganic/organic, carbon-based and hybrid [58]. The inorganic group comprises metal/metal oxide NPs and quantum dots. Organic nanomaterials include polymeric NPs, liposomes and lipid-based NPs that can be used for drug and bioactive delivery [151,152], antimicrobial use, bioimaging and tissue regeneration. Carbon-based nanomaterials comprise carbon black, nanotubes, graphene, nanofibers, nanodots, fullerenes, nano-diamond, carbon onions and carbon rings [58].

Nanoparticle synthesis can be performed by different methods, including physical, chemical and green methods. Physical and chemical approaches usually involve the use of toxic chemicals, which are potentially hazardous to humans and the environment. Compared to conventional methods, biological methods are considered safer and more sustainable for nanomaterial fabrication; therefore, an eco- and environmentally friendly approach for the synthesis of nanoparticles using microorganisms and different plants, commonly referred to as a Green Approach, should be considered [153].

NPs may be used in vaccine production and immunostimulation [154], diagnostic techniques for various diseases, and as disinfectants, growth promoters, antimicrobials (antibacterial, antiviral, antiparasitic and antifungal) and antimycotoxin agents, with one of their most interesting properties being their bacteriostatic activity [69,148].

The use of nanoscale materials as nanobiotics and nano-drug delivery systems can be applied to the production of new antibiotics. Several studies also demonstrated that some NPs, such as AgNPs, can enhance the effect of antibiotics against susceptible and resistant bacteria, and decrease bacterial adhesion and biofilm formation [155,156], revealing a synergy between these compounds. In fact, the use of nanometric size materials can result in greater contact between the compound and the bacteria with increased bioavailability and absorption [157].

To the authors’ knowledge, there are still no licensed nanoparticles for application in the poultry industry. However, studies have been performed with this purpose. The NPs most frequently studied for application in the poultry industry are inorganic NPs, such as copper (CuNPs), zinc (ZnNPs), zinc oxide (ZnONPs), gold (AuNPs), silver (AgNPs) and selenium (SeNPs) NPs. These NPs have been widely explored as antibacterial agents due to their distinctive physicochemical and biological properties [148,149]. Some NPs, especially metallic ones, have antibacterial activity against various bacterial pathogens. Several *in vitro* and *in vivo* studies have shown their inhibitory potential against Gram-positive and Gram-negative bacteria, such as *E. coli*, *S. aureus*, *S.* Enteriditis, *Aeromonas*, *Bacillus*, *Flavobacterium*, *Klebsiella* and *Pseudomonas aeruginosa* [148], but only a few studies have been performed directly with poultry. Table 4 briefly describes the studies carried out on the *in vivo* and *in vitro* application of these NPs against important pathogens in broiler production.

ZnONPs exhibit important antibacterial properties against a wide range of microorganisms, including Gram-positive and Gram-negative bacteria. The properties of ZnONPs (big surface area, biocompatibility, biodegradability, semiconductor behavior and a UV light barrier) contribute to their vast application. These inorganic metal oxide NPs have been used as antimicrobial agents applied via topical creams and animal feed due to their strong bactericidal effect together with small particle size and higher surface energies [150,158]. An *in vitro* study evaluated the antibacterial activity of biologically synthesized ZnONPs against poultry-associated foodborne pathogens such as *Salmonella* spp., *E. coli* and *S. aureus* [149]. The study revealed that ZnONPs exhibit effective antibacterial activity against poultry-associated foodborne pathogens with *S. aureus* being the most susceptible. Moreover, ZnONPs can be used as a feed supplement to reduce the effects of MRSA-induced footpad dermatitis in broiler chickens [148]. Therefore, biosynthesized ZnONPs have great potential to be used as alternative antibacterial agents (nanobiotics) in poultry production to control the gut burden of poultry-associated foodborne pathogens, although further studies are required to evaluate their *in vivo* antibacterial efficacy [149].

AgNPs are one of the most promising nanotechnology products. AgNPs have been reported to have a wide range of antibacterial activities against both Gram-positive and Gram-negative bacteria, including major foodborne pathogens. For example, in 2014, Devi and Bhimba [159] investigated the antimicrobial effect of AgNPs synthesized using the marine algae *Hypnea muciformis*, with the goal of increasing silver nitrate stability. These NPs were tested against several microorganisms, some of them potentially pathogenic to poultry and humans, like *E. coli*, *B. subtilis*, *Klebsiella pneumoniae*, *S. aureus* and *P. aeruginosa*. Currently, there are several commercial products containing AgNPs with broad-spectrum antimicrobial properties. In fact, in human medicine, silver has become a popular additive for many medical applications, such as surgical devices, implants, shunts, catheters and wound dressings [160], and in human dentistry, for preventing the recolonization of bacteria and diminishing biofilm formation [161]. Regarding the poultry industry, AgNPs were shown to promote a reduction in pathogenic bacteria in broiler litter; however, these particles showed no inhibitory activities towards *Campylobacter* in broilers [148]. On the other hand, an *in vivo* study by Salem, Ismael and Shaalan, 2021 [162] evaluated the influence of AgNPs in necrotic enteritis caused by *C. perfringens* in boiler chickens. The authors concluded that these NPs had a positive impact on animals’ gut health integrity and no impact on immune organs. Although AgNPs can accumulate in muscles, further studies are needed to establish the particle concentration and size, the route of administration and the withdrawal time to ensure the safety of chicken meat for human consumption. In fact, Song, Lv, Sheikhahmadi, Uerlings and Everaert, 2017 [163] reported that AgNPs, used as disinfectants in the poultry industry, can have negative effects on chicken health, such as growth and performance impairment. As for other NPs, a trial has been performed to evaluate the effect of the oral administration of Fe_3_O_4_ to chickens challenged with *S.* Enteritidis, and observed a significant decrease in pathogen invasion, as well as reduced inflammatory response associated with infection [69]. Another example is the *in vitro* study by Ali and Bakheet, 2020 [164], which demonstrated that a chitosan NP, a biodegradable polymer, exhibited higher antimicrobial activity against biofilm-forming *E. coli* isolated from cases of omphalitis, in comparison to some antibiotics. A previous report described that zinc, copper and selenium NPs administered *in ovo* at the 18th day of incubation via an amniotic route did not harm the developing embryo, nor affect hatchability [165].

Another NP application, which could prevent the development of antimicrobial drug resistance, is the production of antimicrobial NP carriers, which are usually based on liposomal, solid/lipid, terpenoid, polymeric, dendrimeric and inorganic materials. These carriers can improve the pharmacokinetics of the drugs, extending antimicrobials’ half-life and increasing the distribution volume at the site of infection, resulting in improving antimicrobial potency and eliminating bacterial pathogens at a lower dose. To the authors’ knowledge, there are still no applications of antimicrobial NP carriers available for the poultry production industry [58]. However, some studies have shown some beneficial effects of *Aloe vera* extracts carried by polymeric NPs in improving animals’ growth performance, constituting a possible alternative to reduce antibiotic usage as growth promoters [152]. Another study investigated the administration of nanoencapsulated *Phaleria macrocarpa* fruits extract (NEPM) in chitosan via drinking water. This plant is known for its antimicrobial properties, but has a low efficacy as a diet supplement, due to low solubility, fast degradation and low bioavailability, being thermolabile at body temperature. This study revealed that NEPM contributed to the increase in lactic-acid bacteria in the GIT, modulating the intestinal microbiota and limiting the growth of pathogenic bacteria in broiler chickens [166].

In conclusion, the antibacterial mechanisms of some NPs are still uncertain and their future application depends on complementary studies. Different bacterial strains, action periods, administration routes and NP characteristics have been examined in several studies, which makes the comparison of results difficult, constituting one limitation of the existing research on the antibacterial potential of NPs [167]. Therefore, the potential of NPs’ use as antimicrobial agents in poultry production must be further investigated.

**Table 4:** *In vitro* and *in vivo* studies evaluating antimicrobial effects of NPs in poultry’s health (nd—not determined; na—not applicable).

NP	Target (Gram)	Origin	Trial	Dosage	Effect	Study
ZnO	*Salmonella* spp. (−), *E. coli* (−)*, S. aureus* (+)	*Lactiplantibacillus plantarum* (ex *Lactobacillus plantarum* [131] TA4	*In vitro*	na	Effective antibacterial activity, particularly against *S. aureus*	[149]
Ag	*C. jejuni* (−)	High purity metals and high purity demineralized water	*In vivo*	50 ppm of AgNPs in the drinking water for 30 days	Reduced growth, impaired immune functions and no antibacterial effect on different intestinal bacterial groups	[168]
	*C. perfringens* (+)	Chemical reduction of silver nitrate	*In vivo*	150 µg of AgNPs via crop gavage for 5 days post infection	Reduced colonization of *C. perfringens* in the intestine, positive impact on gut health integrity, and no impact on immune system	[162]
Fe_3_O_4_	*S.* Enteritidis (−)	FeCl_3_ and NaAc.3H_2_O	*In vivo*	Oral 50 mg/kg Fe_3_O_4_-NPs	Pretreatment could significantly decrease the invasion of *S.* Enteritidis in chickens and attenuate morphological changes caused by the infection	[69]
Chitosan	*E. coli* (−)	nd	*In vitro*	na	Antibacterial activity against biofilm-forming strains	[164]
Chitosan encapsulation	*-*	Crab shell and sodium triphosphate pentabasic	*In vivo*	Non-encapsulated (2) and nanoencapsulated extracts of *Aloe vera*, dill and nettle roots (3) in 0.02, 0.025 and 0.05% to starter, grower and finisher diets for 42 days	Nanoencapsulation of dill extract could improve growth performance and can be used as a substitute for AGPs in the diet	[152]
	*-*	nd	*In vivo*	Diet with 2.5% of *Phaleria macrocarpa* fruit extract (2) with nanoencapsulated *Phaleria*, *macrocarpa* fruits extract (NEPM) 2.5% NEPM (3) and with 5.0% NEPM (4)	Diet supplementation with 5.0% of NEMP had positive impact on modulation in the intestinal microbiota and limitation in the growth of pathogenic bacteria	[166]

## 5. Challenges of the Application of These Innovative Compounds

### 5.1. Antimicrobial Peptides

In general, AMPs demonstrate effective activity against microbial pathogens due to their rapid, non-specific action against microorganisms, resulting in a low resistance rate [169]. It is known that bacteria have greater difficulty developing a resistance to AMPs than to antibiotics because most AMPs have the microbial membrane as the main target [170]. However, the overexposure of pathogens to AMPs can generate the development of AMP-resistant strains [169]. Compared with the challenging development of antibiotic resistance, AMP resistance is less worrying due to the lack of horizontal transmission of resistance genes. However, a possible increase in resistance after exposure to AMP-based substances should be expected [171].

There are several mechanisms by which bacteria can develop a resistance to AMPs. First, it is possible that alterations in bacterial membranes, cell walls and cellular metabolism may occur. In the case of membrane modification, the AMP target can be altered, decreasing the interaction of AMPs with membrane components and affecting membrane permeability. Another resistance mechanism results from the modification of bacterial ionic cell potential in specific interaction sites, affecting AMPs’ binding. Furthermore, it is also possible that AMPs activity against bacteria could generate metabolic stress, resulting in the modification of surface structures, such as the bacterial cell envelope, or biofilm production [17,171].

In addition to the possible development of resistances, another challenge inherent to AMPs is their high production costs, in comparison to antibiotics [172]. Lack of stability, susceptibility to enzymatic and pH degradation and low activity under physiological conditions are also recognized problems [169,173].

Due to the aforementioned issues, the application of AMPs to production animals is still limited. The disadvantages described above are responsible for the low number of peptides approved in clinical trials, since the *in vitro* efficacy of these molecules does not always correlate with the *in vivo* one. Nevertheless, AMPs remain a great option to control microbial infections [17].

### 5.2. Bacteriophages

One of the major challenges of bacteriophage therapy is the economic cost related to large-scale phage production that is necessary to cover the needs for the poultry production industry. Torres-Acosta et al., 2019 [174] have developed a bioprocess model for the economic analysis of different manufacture methodologies for bacteriophage products intended for use in poultry production, presenting an innovative approach for the development of phage therapy. This study also concluded that the production titer has a crucial impact on production costs. This parameter still requires optimization and improvement to decrease associated costs [174,175].

Furthermore, bacteriophage therapy can lead to the rapid development of resistance to these antimicrobials [16], which constitutes one of the biggest challenges of its use. Thus, bacterial susceptibility to phage, phage stability and phage efficiency should be monitored during treatment. Moreover, efforts should be undertaken to develop methods for preventing the spread of phage-resistant bacteria [29,176]. Resistance can occur through different mechanisms, such as spontaneous mutations, restriction modification systems and adaptative immunity through the CRISPR-Cas system. Spontaneous mutation is the most common mechanism of phage resistance, conferring resistance by modifying the structure of bacterial surface components, which act as phage receptors and also determine phage specificity. These include lipopolysaccharides (LPS), outer membrane proteins, cell wall teichoic acids, capsules and other bacterial appendices, such as flagella, many of which are involved in bacterial virulence. In fact, phage-resistant bacteria may become less virulent in the case of mutations in genes coding for surface virulence factors [176]. CRISPR-Cas is an adaptive immune system present in most bacteria, which confers protection against infection by phages and other foreign genetic elements [177].

Various strategies may be used to minimize the development of resistance, such as the administration of phage cocktails, instead of isolated phages, aiming to kill the same bacterial strain [178]. As mentioned before, one beneficial characteristic of bacteriophages is their specificity; however, this characteristic can also be detrimental, such as, for example, in the case of infections by *Salmonella*, which has more than 2650 serovars and multiple strains within each serovar, requiring the simultaneous use of multiple phages [16,33].

Another important consideration is that only strong lytic phages with known genomes should be used. Since lysogenic phages incorporate their genetic material into the bacterial genome, they can act as vehicles for HGT between the bacteria of animals or humans via the food chain. Many phage-based products use a combination of different phages, thus enlarging their lytic spectrum [29,179]. Various phage-based preparations have been commercialized and licensed for use in the US. In the EU, however, their implementation is highly regulated. Before therapeutic use, phages should be sequenced to demonstrate the impossibility of a lysogenic cycle and/or the presence of antimicrobial drug resistance genes. The EFSA also encourages research on bacteriophage persistence in foods and on the ability to prevent recontamination with the bacterial pathogen. Currently, there are no regulations or control methods aimed at monitoring bacteriophages and their consumption by humans [180].

### 5.3. Nanoparticles

NPs interact with pathogens via diverse mechanisms, which makes it harder for bacteria to generate resistance to these compounds, in comparison to antibiotics. Thus, numerous mutations are required for bacteria to develop nanoresistance. However, bacteria’s resistance to these substances is still an issue that requires attention [58,161,181]. The mechanisms of bacterial resistance towards NP can be intrinsic or extrinsic. The common resistance mechanisms involve ion efflux pumps, electrostatic repulsion, biofilm formation, enzyme detoxification followed by volatilization and mutations [161]. Nanoresistance changes the shape of bacteria and modulates the expression of membrane proteins, which reverses after the removal of NPs [182]. Although rare, resistance has been reported against inorganic NPs, such as AgNPs, CuNPs, AuNPs and ZnONPs, which can be attributed to increased expression of efflux pumps or alterations in membrane permeability [58,160,161,181,182].

As an example, AgNPs can interact with various targets in the bacterial cell, including the cell membrane, enzymes, proteins, lipids, DNA and plasmids, making the development of resistance to these nanoparticles complex. Despite this, resistance to AgNPs has already been reported [161,181]. Moreover, one of the first reports of bacterial resistance to silver relates to *S.* Typhimurium [183], an important pathogen in poultry production.

Another challenge that NPs face is related to safety. It is crucial that these substances are safe for animal and human application. In poultry production, it is important to first ensure that these substances do not harm the well-being and health of poultry, which are also reflected in the quality of the final product. Secondly, it is important to ensure that their residues are not harmful to humans after consumption of poultry products (eggs and meat). Investigations are now directed to evaluate the toxicity of NPs. Although some studies have shown a low ecotoxicity, others demonstrated some degree of adverse effects of these particles on the tested animals [148]. The toxicity of NPs depend on their concentration, size and charge. Exposure to NPs for long periods can lead to adverse effects on the animals’ immune systems, as well as to their accumulation in organs, such as the liver and spleen. NP aggregates are water soluble and can be harmful to useful bacterial due to low specificity. Metallic NPs can have toxic effects on animals’ tissue cells, as a result of using high concentrations or long-time application, even at a low dose. Thus, the toxicity and safety of these NPs should be evaluated before their addition to animals feed [148,184].

In poultry production, several studies concluded that AgNP overdoses could induce oxidative stress and damage to the liver and intestinal cells of broilers [148,163,185]. The accumulation of AgNPs in chicken liver, yolks, muscle, heart and the bursa of Fabricius is dose dependent and can possibly induce negative consequences in these organs and in the animals’ immune system [148,168]. An *in vivo* study performed by Vadalasetty et al., 2018 [168] investigated the application of AgNPs via drinking water in order to reduce the colonization of *C. jejuni* in challenged broilers. They concluded that, in a concentration of 50 ppm, AgNPs reduced broilers’ growth, impaired immune functions and had no antibacterial effect on several intestinal bacteria, such as *C. jejuni*, lactic acid bacteria, enterococci, *C. perfringens*, *E. coli* and lactose negative enterobacteria. These data may limit the applicability of the use of AgNPs against *C. jejuni* and other pathogens in broiler chickens. The toxicity of other inorganic NPs in poultry has not been well studied, but the inoculation of nanoiron has been found to decrease weight and promote neurologic degeneration, and even mortality, in embryos. Moreover, the *in ovo* injection of CuNPs can have deleterious effects on chicken embryos and hematological/biochemistry alterations in broilers [148].

Another challenge regarding the application of nanoparticles is the use of hazardous chemicals and the high cost of manufacturing, which support the need for the development of alternative methods for nanoparticle production [153]. Nevertheless, some studies have focused on the use of safer and more sustainable approaches for NP production, such as the one by Devi and Bhimba, 2014 [159], which showed that the biosynthesis of these NPs from seaweed can be cost effective and avoid the use of toxic chemicals. As such, further research needs to assess the advantages of the synthesis of these types of materials from sustainable and cost-effective sources.

Overall, information on nanoparticles’ properties, behavior and effects is still scarce, which limits their application in the food industry; however, metal NPs were already incorporated in some human foods and medications, allowing NP-based antibiotic delivery systems and topical application (wounds, dentistry) [184]. Therefore, the potential usage of nanotechnology in poultry production is limited by its possible toxicity and must be investigated in depth.

## 6. Conclusions

Bacterial infections are still a major cause of human and animal morbidity and mortality worldwide. Traditionally, antibiotics were exclusively used for the treatment of bacterial infections; however, their extensive use in human and veterinary medicine and in agriculture resulted in an increase in the selection pressure on bacterial populations in all types of environments [186], leading to the emergence of AMR, which in turn forced a radical change in attitudes in order to preserve the effectiveness of the available compounds and to guarantee global health. It is important that all sectors of the food chain use conventional antimicrobials in a responsible way via a reduced and improved application of these compounds. The effective regulation of antibiotic usage was proven to be highly successful in reducing AMR levels in several European countries [11]. Moreover, the global increase in resistance rates led to the need to investigate the application of alternative antimicrobial products, including in the poultry production setting. These alternatives should be more effective and present new action mechanisms, and include antimicrobial peptides, bacteriophages, probiotics and nanoparticles. Antimicrobial peptides are a large group of substances that can have several benefits in comparison to conventional antibiotics due to their unique action mechanisms and immunomodulatory effects. Bacteriophages also demonstrated a capacity to control some of the most important bacterial agents affecting poultry and, given their specificity, have the ability to not compromise the balance of the animals’ microbiota. Probiotics are already currently used in production animals because of their immunomodulatory activity and intestinal microbiota-modulation ability, both of which are associated with a reduced propensity to infectious disease development and growth-promoting action. Finally, nanoparticles are used not only due to their antimicrobial potential but also because they enhance the action of conventional antibiotics. Despite all their benefits, each of these innovative approaches also have limitations regarding their antimicrobial potential, resistance development, large-scale production costs and safety.

In conclusion, the application of these non-conventional antimicrobials can contribute to a decrease in antimicrobial use and AMR dissemination, with several of them being already in an advanced phase of research for application in human medicine. However, *in vivo* investigations regarding the poultry industry are still scarce and should be supported to slow the development of multidrug-resistant bacteria in these settings.

## Data Availability

The datasets used and/or analyzed during the current study are available from the corresponding author on reasonable request.
